# Adaptation of a Commercial Qualitative BAX^®^ Real-Time PCR Assay to Quantify *Campylobacter* spp. in Whole Bird Carcass Rinses

**DOI:** 10.3390/foods13010056

**Published:** 2023-12-22

**Authors:** Aaron R. Bodie, Dana K. Dittoe, Savannah F. Applegate, Tyler P. Stephens, Steven C. Ricke

**Affiliations:** 1 Meat Science and Animal Biologics Discovery Program, Department of Animal and Dairy Sciences, University of Wisconsin, Madison, WI 53706, USA; 2 Department of Animal Science, University of Wyoming, Laramie, WY 82071, USA; ddittoe@uwyo.edu; 3 Hygiena, 2 Boulden Circle, New Castle, DE 19720, USA; sapplegate@hygiena.com (S.F.A.); tyler@microenvirotech.com (T.P.S.)

**Keywords:** *Campylobacter*, RT-PCR, enrichment-based quantification, enumeration

## Abstract

Poultry is the primary reservoir of *Campylobacter*, a leading cause of gastroenteritis in the United States. Currently, the selective plating methodology using selective agars, Campy Cefex and Modified Charcoal Cefoperazone Deoxycholate agar, is preferentially used for the quantification of *Campylobacter* spp. among poultry products. Due to the specific nature of *Campylobacter*, this methodology is not sensitive, which can lead to skewed detection and quantification results. Therefore, *Campylobacter* detection and quantification methods are urgently needed. The objective was to develop a shortened enrichment-based quantification method for *Campylobacter* (CampyQuant™) in post-chill poultry rinsates using the BAX^®^ System Real-Time PCR assay for *Campylobacter*. The specificity and sensitivity for the detection of *C. jejuni*, *C. coli*, and *C. lari* in pure culture were determined. The BAX^®^ System Real-Time PCR assay consistently detected and identified each species 100% of the time with an enumeration range of 4.00 to 9.00 Log_10_ CFU/mL. Enrichment time parameters for low-level concentrations (0.00, 1.00, and 2.00 Log_10_ CFU/mL) of *Campylobacter* using the BAX^®^ System Real-Time PCR assay were elucidated. It was determined that an enrichment time of 20 h was needed to detect at least 1.00 Log_10_ CFU/mL of *Campylobacter* spp. Using the BAX^®^ System Real-Time PCR assay for *Campylobacter*. As a result, time of detection, detection limits, and enrichment parameters were used to develop the CampyQuant™ linear standard curve using the detected samples from the BAX^®^ System Real-Time PCR assay to quantify the levels in post-chill poultry rinsates. A linear fit equation was generated for each *Campylobacter* species using the cycle threshold from the BAX^®^ System Real-Time PCR assay to estimate a pre-enrichment of 1.00 to 4.00 Log_10_ CFU/mL of rinsates detected. The statistical analyses of each equation yielded an R^2^ of 0.93, 0.76, and 0.94 with a Log_10_ RMSE of 0.64, 1.09, and 0.81 from *C. jejuni*, *C. coli*, and *C. lari*, respectively. The study suggests that the BAX^®^ System Real-Time PCR assay for *Campylobacter* is a more rapid, accurate, and efficient alternative method for *Campylobacter* enumeration.

## 1. Introduction

As *Campylobacter* is among the leading causative agents of gastroenteritis in the US, it is considered a major foodborne pathogen of public health interest. This zoonotic microorganism can be isolated from a wide range of food animals; however, it represents a critical food safety problem for poultry production [1]. Currently, more than 25 species of *Campylobacter* have been identified, and some species such as *Campylobacter jejuni* (*C. jejuni*), *Campylobacter lari* (*C. lari*), and *Campylobacter coli* (*C. coli*), are responsible for most human campylobacteriosis infections [2,3]. The members of this genus are thermophilic, microaerophilic bacterium with optimal temperatures for growth between 37 and 45 °C [4,5,6]. Research has shown that it grows best in an environment with 5% O_2_, 10% CO_2_, and 85% N_2_ [7]. Due to the microaerophilic nature of *Campylobacter*, it is assumed that they are less resilient as they appear to be very sensitive to environmental conditions compared to other foodborne pathogens such as *Salmonella* and *Staphylococcus aureus* [8].

The primary source of human campylobacteriosis originates from poultry products. Approximately 20 to 30% of human infections are linked to the mishandling and consumption of broiler meat, while 50 to 80% may be attributed to chicken, which is a reservoir for *Campylobacter* [9]. Over recent years, there has been a 12% increase in *Campylobacter* cases reported between 2015 and 2017 [10]. This can partly be attributed to the fact that the epidemiology of campylobacteriosis in poultry is still poorly understood [11]. As a result, the rapid and reliable detection and quantification of *Campylobacter* directly from poultry samples remains a challenge. 

Currently, the identification and quantification of *Campylobacter* spp. relies on culture-based methods and phenotyping [12]. These conventional methods include selective plate enumeration, optical microscopy, and fluorescence optical density measurements that lead to an elevated level of specificity; however, these methods do possess disadvantages. When culturing *Campylobacter* spp., in stressful environments, cells can become viable but nonculturable (VBNC) and further complicate enumeration [13]. Detection and quantitation can be time-consuming, requiring prolonged incubation periods, and it can take several days to retrieve the results [14,15]. 

Molecular assays such as real-time polymerase chain reaction (RT-PCR) provide an alternative to microbiological methods of *Campylobacter* detection and quantification [15]. The real-time PCR assay is a rapid and specific nucleic acid amplification method for detection with advantages in terms of turnaround time, specificity, and sensitivity. The BAX^®^ System Real-Time PCR assay for *Campylobacter* is a commercial PCR assay system approved by the Association of Official Agricultural Chemists (AOAC) International for the detection of *Campylobacter* in food and environmental samples. The BAX^®^ System amplifies an approximately 400-base-pair species-specific genomic region [16]. The BAX^®^ System Real-Time PCR assay is specific to the multiplex detection and quantification of *C. jejuni*, *C. lari*, and *C*. *coli*. The assay does not amplify sequences of other *Campylobacter* species or react with non-*Campylobacter* bacteria prominent in poultry samples, such as *Salmonella*, *Escherichia coli*, and *Lactobacillus* spp. [17]. The BAX^®^ System Real-Time PCR assay minimizes problems common to phenotypic and antibody-based methods, such as cross-reaction with related organisms [18]. Thus, it provides the opportunity for specific screening of *Campylobacter* in poultry samples [18].

Therefore, the objective of this study was to develop and optimize a rapid quantification method for *Campylobacter* species (CampyQuant™) in post-chill whole bird carcass rinsates (WBCR) using the BAX^®^ System Real-Time PCR assay for *Campylobacter*. As such, the first objective was to determine the optimal enrichment media, buffered peptone water (BPW), and 2× blood-free Bolton broth (2× BFBB), to optimize the parameters of the BAX^®^ System Real-Time PCR assay. As BPW is a widely used enrichment media by the United States Department of Agriculture–Food Safety Inspection Service (USDA-FSIS) for foodborne pathogen enrichment (*Salmonella*), the use of it as an enrichment media in the current assay would have allowed for the identification and quantification of *Campylobacter* alongside several other pathogens [19]. The 2× BFBB was chosen as the other enrichment media due to its use by the USDA-FSIS as the primary enrichment media for *Campylobacter* [20]. After determining the optimal enrichment media, the detection limit was determined to mitigate enrichment parameters and establish a detection limit for each *Campylobacter* species using the BAX^®^ System Real-Time PCR assay for *Campylobacter*. Lastly, the media comparison and enrichment time of detection parameters were used to develop a standard linear curve using the detected samples from the BAX^®^ System Real-Time PCR assay. The development of this standard curve, in turn, allows the BAX^®^ System to act as a potential quantification method for *C. jejuni*, *C. coli*, and *C. lari* in poultry rinsates.

## 2. Materials and Methods

### 2.1. Campylobacter spp. Pure Cultures

*Campylobacter* species *C. jejuni* American Type Culture Collection^®^ (ATCC^®^) 700819, *C. coli* ATCC^®^ BAA—1061™, and *C. lari* ATCC^®^ BAA—1060™ were used for this study. The ATCC^®^ strains utilized in the current study were chosen due to the extensive data available on their ecology, functionality, and genetic and biochemical characteristics. To obtain precultures for the growth experiments, frozen stock cultures (−80 °C) of each *Campylobacter* species (*C. jejuni*, *C. coli*, and *C. lari*) were plated on modified Charcoal Cefoperazone Deoxycholate Agar (mCCDA; Himedia, Mumbai, India) and incubated under microaerophilic conditions (5% O_2_, 10% CO_2_, and 85% N_2_) using the Anoxomat^®^ III system (Advanced Instruments, Norwood, MA, USA) at 42 °C for 48 h. After incubation, one isolated colony was inoculated in 10 mL of blood-free Bolton broth (BFBB; Criterion, Hardy Diagnostics, Santa Maria, CA, USA), followed by incubation under microaerophilic conditions at 42 °C for 48 h using the Anoxomat^®^ III system. The cell density of the inoculum used throughout the study was 9 Log_10_ CFU/mL, as determined by spread plating on mCCDA plates. 

### 2.2. Objective 1: Determining Optimal Enrichment Media and Enumeration Range of BAX^®^ System Real-Time PCR Assay

After 48 h of growth, 1 mL of *Campylobacter jejuni, coli*, and *lari* cultures were individually transferred to 19 mL of fresh 2× BFBB with 2× the antibiotic supplement (Cefoperazone 40 mg/L, Vancomycin 40 mg/L, Trimethoprim 40 mg/L, and Cycloheximide 50 mg/L) and BPW and incubated under microaerophilic conditions (5% O_2_, 10% CO_2_, and 85% N_2_) using one anaerobic pack sachet (Thermofisher Scientific, Waltham, MA, USA) for 24 h. Following 24 h incubation, a 10-fold dilution to 10^−6^ was performed on each medium using 9 mL of the respective medium (2× BFBB or BPW) and 1 mL of the 24 h *Campylobacter* species inoculum. Samples were spread plated using 100 μL of diluent on Modified Charcoal Cefoperazone Deoycholate Agar (mCCDA) to determine the concentration of *Campylobacter*. Additionally, *Campylobacter* in all media samples and dilutions was detected using the BAX^®^ System Real-Time PCR assay for *Campylobacter* (KIT2018, Hygiena, Camarillo, CA, USA) according to the manufacturer’s instructions. The experiment was repeated three times with three technical replications per medium (N = 54 total number of samples; media: k = 2; species: k = 3; biological replicates: n = 3; trial: t = 3; Appendix A—Figure A1). A standard curve was obtained from the amplification (cycle threshold; CT) of the genomic DNA extracted from the dilutions (1:10) of the pure culture samples using the BAX^®^ System.

### 2.3. Objective 2: Time of Detection of Campylobacter spp. in Enriched Poultry Rinsates

Poultry post-chill rinsates were obtained from a commercial poultry producer in the southeastern United States and shipped via overnight courier to the Meat Science and Animal Biologics Discovery (MSABD) building at the University of Wisconsin-Madison (UW-Madison, Madison, WI, USA) in an insulated shipping cooler with frozen gel packets (ULINE, Pleasant Prairie, WI, USA). Upon arrival at MSABD, rinsates were stored at −20 °C until use. 

No more than 24 h prior to the onset of the experiment, rinsates were thawed and combined to create bulk rinsates to reduce the variation between biological rinsate samples. Additionally, prior to the study, the bulk rinsate was screened for the presence of *Campylobacter* using the BAX^®^ System Real-Time PCR assay for *Campylobacter* (KIT2018, Hygiena, Camarillo, CA, USA) according to the manufacturer’s instructions to determine if there was a significant presence of indigenous *Campylobacter*. Only bulk rinsates confirmed free of *Campylobacter* were utilized for the remainder of the study.

The pH of the bulk rinsate was measured with a SympHony pH meter and probe (VWR International, Radnor, PA, USA) to determine if the rinsates needed to be neutralized (7 pH) since many poultry processors use processing aids that alter the pH and this could impact downstream microbiological inferences [21]. All bulk homogenate rinsates in the current study had a pH of 7 ± 0.2 and did not require neutralization using sodium thiosulfate, sodium bicarbonate, and soy lecithin [21].

From the bulk post-chill poultry rinsates, 30 mL was aliquoted into 24 oz sterile Whirl-Pak (Nasco, Fort Atkinson, WI, USA) bags and inoculated at a targeted 0.00, 1.00, and 2.00 Log_10_ CFU/mL of *C. jejuni* (ATCC^®^ 700819™), *C. coli* (ATCC^®^ BAA—1061™), or *C. lari* (ATCC^®^ BAA—1060™) (N = 216 total number of samples; k = 3 number of treatments; n = 24 number of samples per treatment group per timepoint; 3 timepoints; 8 technical replicates/bio replicate; 1 non-inoculated sample/enrichment time; Appendix A—Figure A2). Subsequently, 30 mL of pre-warmed (42 °C) 2× BFBB with 2× the antibiotic supplement was added to each sample and incubated at 42 °C for either 16, 18, or 20 h, respectively [20].

When this research was performed, 2× BFBB with 2× antibiotic supplements was recommended by the USDA-FSIS to enrich *Campylobacter* in poultry rinsates [20,22]. Per the USDA-FSIS, samples were incubated under microaerophilic conditions using one anaerobic pack sachet (ThermoFisher Scientific, Waltham, MA, USA) inside a 7 L anaerobic box (Mitsubishi, New York City, NY, USA). At each enrichment time, samples were removed from the incubator. *Campylobacter* presence and quantity were determined the BAX^®^ System Real-Time PCR assay for *Campylobacter* (KIT2018, Hygiena, Camarillo, CA, USA) according to the manufacturer’s package insert, with 8 technical replicates per biological replicate (n = 3). 

### 2.4. Objective 3: Validation and Standard Curve Development of Quantification of BAX^®^ System Real-Time PCR Assay 

After elucidating the enrichment time parameters for the limit of detection using the BAX^®^ System Real-Time PCR assay, a standard curve was developed for each species. Bulk post-chill WBCR were prepared as previously described in Objective 2. The initial concentrations of *C. jejuni*, *C. coli*, and *C. lari* before dilutions were 9.01, 9.13, and 9.05 Log_10_ CFU/mL, respectively.

For each serovar, *C. jejuni* (ATCC^®^ 700819™), *C. coli* (ATCC^®^ BAA—1061™), or *C. lari* (ATCC^®^ BAA—1060™), 30 mL of the bulk post-chill WBCR was aliquoted into 24 oz Whirl-Pak bags (N = 39). Samples were either uninoculated (n = 1) or inoculated with 1.00, 2.00, 3.00, or 4.00 Log_10_ CFU/mL (n = 3 at each inoculation level) of *Campylobacter* spp. Pre-warmed (42 °C) 2× BFBB (30 mL) with 2× antibiotic supplements was aseptically added to the inoculated post-chill WBCR (30 mL). Enrichments prior to incubation were enumerated on Campy Cefex to confirm inoculation level. Samples were subsequently incubated under microaerophilic conditions using one anaerobic pack sachet (ThermoFisher Scientific, Waltham, MA, USA) inside a 7 L anaerobic box (Mitsubishi, New York City, NY, USA) at 42 °C for 20 h. Campy Cefex plates were used throughout the study to retain consistency with regulatory and industry standards [20] (USDA-FSIS, 2021). Plated samples were incubated for 48 h using the Anoxomat^®^ III system (Advanced Instruments, Norwood, MA, USA). 

Enriched samples were tested in quintuplet with the BAX^®^ System Real-Time PCR assay for *Campylobacter* (KIT2018, Hygiena, Camarillo, CA, USA) according to the manufacturer’s package insert. The generation of a lysate and subsequent amplification of the genomic DNA of the inoculated samples was performed using the BAX^®^ standard protocols for poultry WBCR [23]. After the 20 h incubation, 5 µL of the samples was run on the BAX^®^ System Real-Time PCR to obtain a CT value. A linear regression curve was generated using CT values detected from the PCR and inoculated sample concentration. 

### 2.5. Statistical Analyses

#### 2.5.1. Objective 1: Determining Optimal Enrichment Media and Enumeration Range of BAX^®^ System Real-Time PCR Assay

All statistical analyses for this study were performed in JMP 14.0 (SAS Institute Inc., Cary, NC, USA). Among the pure cultures, each dilution series of the sample was statistically analyzed using linear regression analysis to determine if there was a relationship between CT value and *Campylobacter* detection in the BAX^®^ System Real-Time PCR. After linear regression, a Mann–Whitney U test was used to determine significant differences within species and the detection limit between different media for each species in the BAX^®^ System Real-Time PCR. Quantification metric parameters including specificity, sensitivity, efficiency, accuracy, positive and negative likelihood ratios (PLR, NLR), prevalence, positive predictive value (PPV), and negative predictive value (NPV) were calculated for each species and media using the following equations:

$$Specificity=1-\frac{false\;positives\;}{true\;negatives+false\;positives}$$

$$Sensitivity=1-\frac{false\;negatives}{true\;positives+false\;negatives}$$

$$Efficiency=-1+10^{(-1/slope)}$$

$$Accuracy=\left(sensitivity\times prevalence\right)+(specificty\times \left(1-prevalence\right))$$

$$PLR=\frac{sensitivity}{1-specificity}$$

$$NLR=\frac{1-sensitivity}{specificity}$$

$$Prevalence=\frac{positive\;samples}{total\;samples}$$

$$PPV=\frac{true\;positives}{true\;positives+false\;positives}$$

$$NPV=\frac{true\;negatives}{true\;negatives+false\;negatives}$$

The main effect and interaction of media and species were evaluated for the BAX System quantification metric parameters such as sensitivity, accuracy, prevalence, NLR, and NPV using a Mann–Whitney U test. Other metric parameters were not statistically analyzed because the numerical values were the same, resulting in no differences. Further analysis was conducted on quantification metric parameters to determine differences between *Campylobacter* species (*C. jejuni*, *C. coli*, and *C. lari*) in 2× BFBB and BPW and between the enrichment media in *Campylobacter* species. Pairwise differences were analyzed using an χ^2^ analysis. Significant differences were considered at *p* ≤ 0.05. 

#### 2.5.2. Objective 2: Time of Detection of *Campylobacter* spp. in Enriched WBCR

To determine the appropriate time needed for *Campylobacter* detection in the BAX^®^ System Real-Time PCR, three time points were used, 16, 18, and 20 h, based on the growth kinetics of the three isolates used in this study [24]. PCR quantification metric parameters, sensitivity, specificity, PLR, NLR, prevalence, PPV, NPV, and accuracy, were calculated for each species and time point. Furthermore, the quantification metrics were analyzed for main effect and interactions using a Mann–Whitney U test. Pairwise differences comparing significances between the time (16, 18, and 20 h) of *Campylobacter jejuni*, *coli*, and *lari* and the species *Campylobacter* species (*C. jejuni*, *C. coli*, and *C. lari*) at 16, 18, and 20 h of enrichment were evaluated using an χ^2^ Analysis. Significant differences were considered at *p* ≤ 0.05.

#### 2.5.3. Objective 3: Validation and Standard Curve Development of Quantification of BAX^®^ System Real-Time PCR Assay

In poultry WBCR, for each *Campylobacter* species, 1.00 to 4.00 Log_10_ CFU/mL of sample was statistically analyzed using a linear regression model to determine the relationship with CT values from the BAX^®^ System and plate counts. Additionally, PCR quantification metric parameters, sensitivity, accuracy, prevalence, NLR, and NPV, were analyzed among CampyQuant™ and Campy Cefex plates. *Campylobacter* WBCR samples’ main effects and comparisons quantified using Campy Cefex and CampyQuant™ were explored for sensitivity, accuracy, prevalence, NLR, and NPV using a Mann–Whitney U test. Pairwise differences were determined using an χ^2^ analysis. An ANCOVA test was run to investigate if there was a significant different linear slope between *C. jejuni*, *C. coli*, and *C. lari* after 20 h of enrichment in poultry rinsate samples. Significant differences were considered at *p* ≤ 0.05.

## 3. Results

### 3.1. Objective 1: Determining Optimal Enrichment Media and Enumeration Range of BAX^®^ System Real-Time PCR Assay

The first objective aimed at determining the optimal enrichment media of the BAX^®^ System Real-Time PCR *Campylobacter* (*jejuni*, *coli*, *lari*) assay. To accomplish this objective, two standard media, 2× BFBB and BPW, were inoculated with varying levels of either a 24 h culture of *Campylobacter jejuni*, *coli*, or *lari* that were grown in respective media and the cycle thresholds (CT) were determined using the BAX^®^ System Real-Time PCR *Campylobacter* (*jejuni*, *coli*, *lari*) assay. The sensitivity, specificity, efficiency, positive likelihood ratio (PLR), negative likelihood ratio (NLR), prevalence, positive predictive value (PPV), negative predictive value (NPV), and accuracy of the detection of *Campylobacter* species within media and differences of media within species, as well as their interaction, were analyzed. Lastly, the enumeration range and linear fit of *Campylobacter jejuni*, *coli*, and *lari* was compared between 2× BFBB and BPW, with the slopes being compared using ANCOVA.

#### 3.1.1. Optimal Enrichment Media

There was no interaction between *Campylobacter* species and media on quantification quality parameters (*p* > 0.05), except for accuracy (*p* < 0.05; Table 1). The accuracy of detection of *Campylobacter* was highest among *C. jejuni* and *C. coli* enriched in 2× BFBB (93.8 and 96.3%) compared to *C. jejuni*, *coli*, and *lari* enriched in BPW (81.9, 79.0, and 84.0%). The accuracy of detection of *C. lari* when enriched in 2× BFBB was not different than when enriched in BPW (89.8 and 84.0%). There was not a main effect of *Campylobacter* species on PCR quantification quality metrics; however, there was a main effect of media on the sensitivity, prevalence, NLR, NPV, and accuracy (*p* < 0.05; Table 1). The sensitivity (92.5 and 78.3%), prevalence (87.2 and 70.5%), NLR (7.6 and 26.7%), NPV (40.74 and 14.1%), and accuracy (93.3 and 81.6%) were higher among *Campylobacter* species grown and inoculated in 2× BFBB than in BPW (*p* < 0.05). Additionally, the impact that media had on the performance characteristics within individual *Campylobacter* species and the effect of species on the performance within each media is defined in Tables S1–S3. Similar effects were seen within these analyses as in the full model and for brevity will not be discussed in the text. 

#### 3.1.2. Enumeration Range and Linear Fit

After growth in 2× BFBB or BPW, *Campylobacter jejuni*, *coli*, and *lari* were serially diluted in respective media and the cycle thresholds (CT) were determined using the BAX^®^ System Real-Time PCR *Campylobacter* (*jejuni*, *coli*, *lari*) assay. The 24 h culture was also plated onto mCCDA to confirm the inoculation level to determine linear fit and to compare the slopes between the two media, 2× BFBB or BPW. Overall, the initial concentration of *C. jejuni*, *coli*, and *lari* were 8.5, 9.0, and 8.8 and 8.6, 8.5, and 7.5 Log_10_ CFU/mL in 2× BFBB and BPW, respectively. Linear curves were developed within the range of the lower limit of detection in BPW (Table S4). The lower limit of detection was determined as the initial inoculation level producing a CT value within range (<40) of the BAX^®^ System. The lower limit of detection of *C. jejuni*, *coli*, and *lari* was 3.03, 2.93, and 3.13 Log_10_ CFU/mL in 2× BFBB, whereas the lower limit was 5.60, 5.50, and 4.50 Log_10_ CFU/mL in BPW (Table S4). As such, across *Campylobacter* species, only four dilutions in BPW ranging from 5.6 to 8.6, 5.5 to 8.5, and 4.5 to 7.5 Log_10_ CFU/mL were able to be utilized for linear fit.

**Figure 1:**
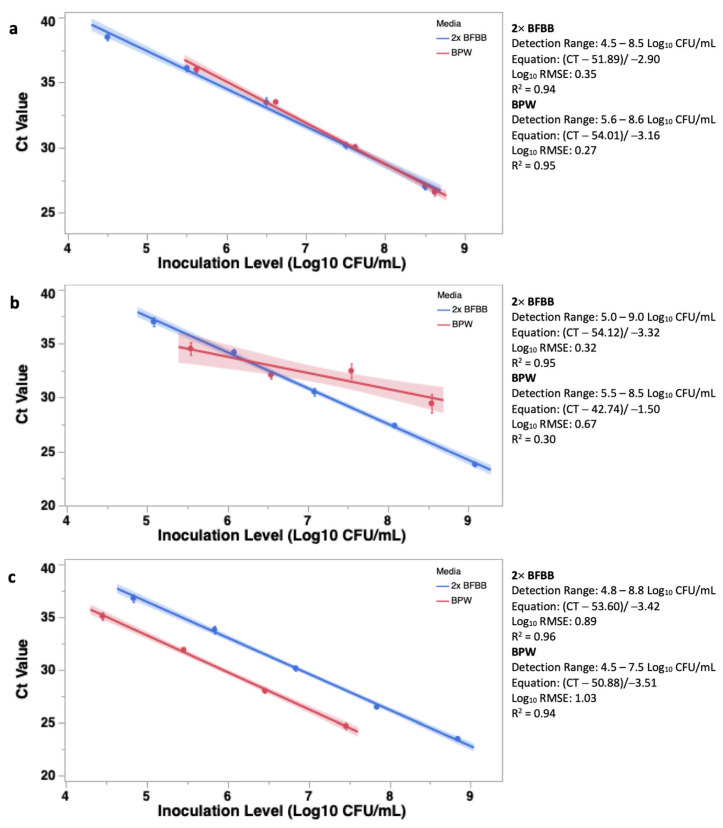
The linear response of the cycle threshold (CT) to the inoculation of *Campylobacter jejuni* (**a**), *coli* (**b**), and *lari* (**c**) in either 2× blood-free Bolton’s broth (2× BFBB) or Buffered Peptone Water (BPW). The CT values were obtained using the BAX^®^ System Real-Time PCR assay. There was a difference between the detection in 2× BFBB and BPW of all three *Campylobacter* species (Mann–Whitney U: *p* < 0.05), as well as a difference between the slopes generated from 2× BFBB and BPW (ANCOVA: F < 0.01).

There was a significant difference between the slopes of *C. jejuni* detected in 2× BFBB and BPW (−2.90 and −3.16, F < 0.01; Figure 1a). However, the linear fit (R^2^ = 0.94 and 0.95) and Log_10_ RMSE (0.35 and 0.27) were similar (Figure 1a). There were also differences between the slopes of *C. coli* inoculated into 2× BFBB and BPW, with the slope being greater in 2× BFBB (−3.32 and −1.50, F < 0.01; Figure 1b). The fit of the data (R^2^ = 0.95 and 0.30) and Log_10_ RMSE (0.32 and 0.67) were stronger among the linear curves belonging to 2× BFBB (Figure 1b). There was also a difference in the slopes generated when *C. lari* was inoculated into 2× BFBB or BPW, with the slope of BPW being greater than that of 2× BFBB (−3.42 and −3.51; F < 0.01; Figure 1c). The linear fit (R^2^ = 0.96 and 0.94) and Log_10_ RMSE (0.35 and 0.27) were similar (Figure 1c).

### 3.2. Objective 2: Time of Detection of Campylobacter spp. in Enriched Poultry WBCR

The results of the first objective indicated that the optimal enrichment media is 2× BFBB. However, samples needed to be within the enumerable range to achieve optimal quantification outcomes, as identified in objective 1. As such, the samples would need to be enriched to be within the quantifiable range. Therefore, the second objective was to determine the time of enrichment necessary for the detection of low *Campylobacter jejuni*, *coli*, and *lari* concentrations in commercial poultry WBCR. As the WBCR were collected from a commercial poultry operation post-chill, the impact of the indigenous microbiota on the detection of low *Campylobacter jejuni*, *coli*, and *lari* concentrations using the BAX^®^ System Real-Time PCR assay could be explored. Post-chill WBCR (7.04 pH) were inoculated with a target concentration of 0.00, 1.00, and 2.00 Log_10_ CFU/mL of individual *Campylobacter* species and enriched in 2× BFBB for either 16, 18, or 20 h. At each time point, the *Campylobacter* within enriched and inoculated poultry WBCR were subjected to the BAX^®^ System Real-Time PCR assay. The PCR quality parameter metrics were determined and statistically compared.

The target inoculation levels of 0.00, 1.00, and 2.00 Log_10_ CFU/mL of individual *Campylobacter* species were confirmed via plating on mCCDA and were determined to be 0.58, 1.58, and 2.58 Log_10_ CFU/mL for *C. jejuni*, 0.17, 1.17, and 2.17 Log_10_ CFU/mL for *C. coli*, and 0.03, 1.03, and 2.03 Log_10_ CFU/mL for *C. lari*. The prevalence of individual inoculation levels was determined and increased with Log_10_ CFU/mL concentration and enrichment time (Table S5). At 20 h of enrichment, both the target inoculation of 1 and 2 Log_10_ CFU/mL had a prevalence greater than 88% among the inoculated samples (Table S5). 

To further delineate the effect of enrichment time on the PCR performance, the main effect and interaction of enrichment time (16, 18, and 20 h) and *Campylobacter* species (*C. jejuni*, *coli*, and *lari*) was determined (Table 2). There was an interaction between the enrichment time and *Campylobacter* species on PCR performance metrics, sensitivity, NLR, NPV, and accuracy (*p* < 0.05; Table 2). The sensitivity was the greatest at 20 h of enrichment among *C. jejuni* inoculated rinsates (94.4%) and was not different from the sensitivity of *C. jejuni* detection at 18 h of enrichment (86.1%). The sensitivities of 20 h of enrichment of *C. coli* and *C. lari* (63.9 and 72.2%) were not different from one another or different from *C. jejuni* at 16 h (66.7%). The sensitivity of *C. coli* at 16 and 18 h of enrichment (14.6 and 25.7%) and *C. lari* at 16 h of enrichment were the lowest among samples (13.9%). The NLR was lowest at 20 h of enrichment among *C. jejuni* inoculated samples (5.6%) which was not different than *C. jejuni* inoculated samples enriched for 18 h (13.9%). The NLR of *C. lari* inoculated samples enriched for 20 h (27.7%) was not different from those inoculated with *C. jejuni* and enriched for 16 or 18 h (33.3 and 13.9%) or those inoculated with *C. coli* and enriched for 20 h (36.1%). The NPV was highest among those inoculated with *C. jejuni* and enriched for 20 h (44.4%) and was significantly different than all other samples. Those inoculated with *C. jejuni* and enriched for 18 h (17.0%) had a higher NPV than those inoculated with *C. lari* and enriched for 16 h (5.1%). The accuracy was highest among samples inoculated with *C. jejuni* and enriched for 18 and 20 h (71.4 and 78.1%) compared to all other samples. The accuracy was significantly lower among those inoculated with *C. coli* (3.0 and 8.6%) and *C. lari* (1.2 and 27.3%) and enriched for 16 or 18 h, as compared to those enriched for 20 h (39.2 and 50.4%).

Although there was not a significant interaction between the enrichment time and *Campylobacter* species, there was a main effect for both (Table 2, *p* < 0.05). The prevalence increased as the enrichment time progressed, with 20 h (73.8%) being greater than both 16 and 18 h (34.2 and 55.1%). Among the three species of *Campylobacter*, the highest prevalence was calculated among samples inoculated with *C. jejuni* (79.1%), as compared to *C. coli* and *C. lari* (37.3 and 46.7%). There was no difference between the prevalence calculated among samples inoculated with *C. coli* and *C. lari* (37.3 and 46.7%). 

Additionally, the impact that the enrichment time had on the performance characteristics within individual *Campylobacter* species and the effect of species on the performance within each enrichment time are defined in Tables S5–S7. Similar effects were seen within these analyses as in the full model and for brevity will not be discussed in the text. In summation, 20 h enrichment resulted in better sensitivity, accuracy, prevalence, and NLR and NPV values for all *Campylobacter* species, as compared to 16 and 18 h enrichment (Table 2 and Tables S5–S7). These results suggest that after 20 h of enrichment in poultry rinsates, *Campylobacter* could be detected from low concentrations of 1.00 and 2.00 Log_10_ CFU/mL.

### 3.3. Objective 3: Validation and Standard Curve Development of Quantification of BAX^®^ System Real-Time PCR Assay 

After determining that the enrichment time needed for low concentrations of *Campylobacter* to be detected in the BAX^®^ System Real-Time PCR assay was 20 h of incubation, a CampyQuant™ standard curve was developed for *Campylobacter* quantification in poultry WBCR (7.03 pH) using a linear regression model. The resulting linear fit equation generated from the CT value from the amplification of *Campylobacter* within the inoculated rinsates (1.00 to 4.00 Log_10_ CFU/mL) that were enriched for 20 h was subsequently employed. The sensitivity, prevalence, NLR, NPV, and accuracy of the BAX^®^ System Real-Time PCR assay were compared to the current standard quantification method using traditional spread plating on Campy Cefex agar where the main effect and interaction of the quantification method and *Campylobacter* species were determined. 

#### 3.3.1. CampyQuant™ Linear Fit and Subsequent Equation

The linear fit and resulting equation was generated for each *Campylobacter* species using the CT values from the BAX^®^ System to estimate the pre-enrichment Log_10_ CFU/mL of WBCR. The linear equation was used to simulate the logarithmic growth of the bacteria in order to estimate the pre-enriched *Campylobacter* levels in poultry carcass rinses. All three *Campylobacter* species had a relation between data and the linear equation, with an R^2^ of 0.934 (1), 0.758 (2), and 0.943 (3), respectively, with *C. jejuni* and *C. lari* exhibiting a stronger relationship than *C. coli*. The Log_10_ RMSE was also within 1 Log_10_ CFU/mL at 0.64 (1), 1.09 (2) and 0.81 (3) Log_10_ RMSE. The equations generated were as follows:

(1) $$C.\;jejuni\;\left({Log}_{10}\;CFU/mL\right)=\frac{CT-41.64}{-2.281}$$

(2) $$C.\;coli\;\left({Log}_{10}\;CFU/mL\right)=\frac{CT-39.98}{-1.896}$$

(3) $$C.\;lari\;\left({Log}_{10}\;CFU/mL\right)=\frac{CT-40.75}{-3.03}$$

**Figure 2:**
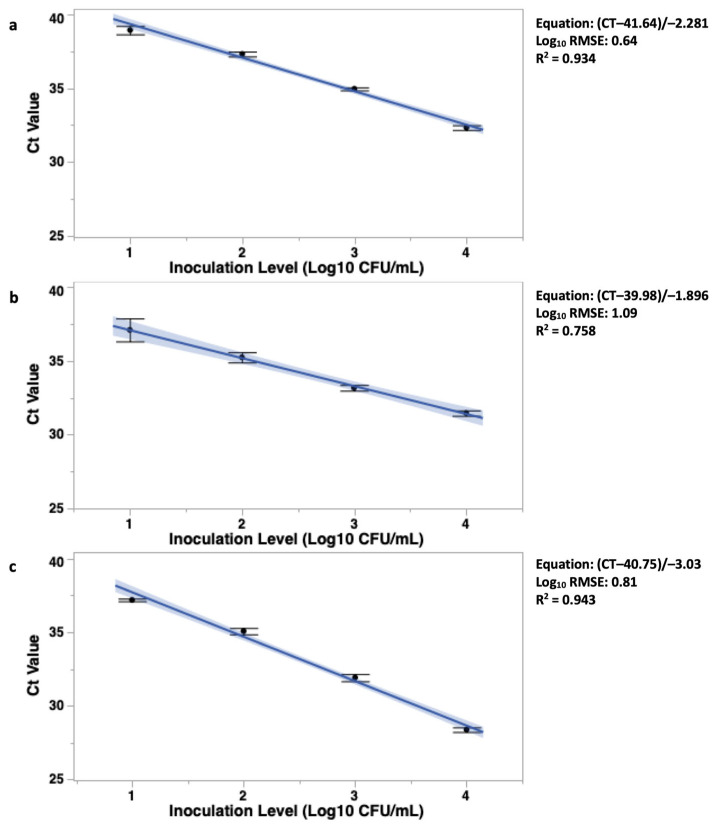
Development of standard curves of *Campylobacter jejuni* (**a**), *coli* (**b**), and *lari* (**c**) to be used for enrichment-based quantification of these three *Campylobacter* species with enumerable ranges from 1.00 to 4.00 Log_10_ CFU/mL. The CT values were obtained using the BAX^®^ System Real-Time PCR assay. There was a difference between the slopes generated from the three species (ANCOVA: F < 0.01).

Also, using CampyQuant™, an ANCOVA test was used to investigate if there was a significant difference between the linear slopes of curve generated from *C. jejuni*, *C. coli*, and *C. lari* after 20 h of enrichment in poultry rinsate samples. There were differences among the slopes of the three *Campylobacter* species (F < 0.05; Figure 2). The *C. coli* linear slope was the least horizontal at −1.89, followed by *C. jejuni* with a linear slope of −2.28. The lowest linear slope was *C. lari*, at −3.03. 

#### 3.3.2. *Campylobacter* spp. Levels from Each Quantification Method 

Using the equations generated from linear regression, CampyQuant™ estimates of *C. jejuni*, *coli*, and *lari* were generated and compared to the spread-plated estimates of the three species based on the industry standard Campy Cefex (Table 3). The main effect and subsequent interaction of quantification method and species was explored at individual inoculation levels (Table 3). There was an interaction of quantification method and *Campylobacter* species on the estimated *Campylobacter* levels at each inoculation level (*p* < 0.05; Table 3). At 1.00 Log_10_ CFU/mL (*p* < 0.05), *C. jejuni* detection in Campy Cefex (1.64 Log_10_ CFU/mL) was significantly higher than *C. jejuni* detected with CampyQuant™ (1.18 Log_10_ CFU/mL) or *C. lari* detected with either CampyQuant™ or Campy Cefex (1.18 and 0.70 Log_10_ CFU/mL). There were no differences in detection of *C. jejuni* on Campy Cefex (1.64 Log_10_ CFU/mL), *C. coli* on CampyQuant™ (1.212 Log_10_ CFU/mL), or *C. coli* on Campy Cefex (1.26 Log_10_ CFU/mL). At 2.00 Log_10_ CFU/mL, *C. jejuni* enumerated on Campy Cefex plates (2.18 Log_10_ CFU/mL) was significantly higher than *C. jejuni* quantified using CampyQuant™ (1.89 Log_10_ CFU/mL), *C. coli* quantified on Campy Cefex plates (1.74 Log_10_ CFU/mL), and *C. lari* quantified on both CampyQuant™ (1.88 Log_10_ CFU/mL) and Campy Cefex plates (1.50 Log_10_ CFU/mL). Moreover, there was no difference between quantification of *C. jejuni* on Campy Cefex plates (2.18 Log_10_ CFU/mL) and *C. coli* enumeration using CampyQuant™ (2.09 Log_10_ CFU/mL). At 3 Log_10_ CFU/mL, *C*. *coli* quantified on Campy Cefex plates (3.23 Log_10_ CFU/mL) was the highest concentration seen and significantly different from *C. coli* using CampyQuant™ (2.74 Log_10_ CFU/mL) and *C. lari* using either CampyQuant™ (2.92 Log_10_ CFU/mL) or Campy Cefex plates (2.68 Log_10_ CFU/mL). No differences were seen between *C. coli* using Campy Cefex plates and *C. jejuni* enumerated using either CampyQuant™ (2.93 Log_10_ CFU/mL) or Campy Cefex plates (3.05 Log_10_ CFU/mL). At 4.00 Log_10_ CFU/mL, the highest concentration used in the study, *C. coli* quantified using Campy Cefex (4.13 Log_10_ CFU/mL) was significantly higher than *C. coli* with CampyQuant™ (3.90 Log_10_ CFU/mL) and *C. lari* enumerated using Campy Cefex plates (3.55 Log_10_ CFU/mL). When compared to *C. coli* using Campy Cefex, no differences were observed against *C. jejuni* quantified with either CampyQuant™ or Campy Cefex (4.08 and 4.03 Log_10_ CFU/mL) and C. *lari* quantified using CampyQuant™ (4.09 Log_10_ CFU/mL). Additionally, the impact that the quantification method had on the estimation of *Campylobacter* within individual *Campylobacter* species is defined in Table S8. Similar effects were seen within these analyses as in the full model and for brevity will not be discussed in the text. 

#### 3.3.3. Performance Parameters

Quantification metric parameters (sensitivity, prevalence, NLR, NPV, and accuracy) were statistically compared to determine the main effect and interaction of the quantification method (CampyQuant™ vs. Campy Cefex) and *Campylobacter* species on the aforementioned metrics (Table 4). The Mann–Whitney U test showed no interactions between the quantification method and *Campylobacter* species (*p* > 0.05; Table 4). A main effect was seen between the three *Campylobacter* species for NPV (*p* < 0.05; Table 4). *C. jejuni* had the highest NPV (CampyQuant™ 34.4%; Campy Cefex 30.5%) among species, followed by *C. lari* (CampyQuant™ 26.1%; Campy Cefex 27.8%) and, lastly, *C. coli* (CampyQuant™ 20.6%; Campy Cefex 15.9%). Additionally, the impact the quantification method had on the performance parameters within individual *Campylobacter* species and the effect of species on performance parameters within each quantification method are detailed in Tables S9–S11. Similar effects were seen within these analyses as in the full model and for brevity will not be discussed in the text. 

## 4. Discussion

In 2016, the USDA-FSIS proposed the performance standards for *Campylobacter* and *Salmonella* in an effort to mitigate pathogen presence in poultry carcasses [25]. Currently, prevalence-based data are used to determine process controls. However, little insight is provided. In addition, regulatory baselines and the literature have, for the most part, utilized direct plating to estimate *Campylobacter* concentrations in poultry products. Currently, limited published literature evaluates the efficacy of various enrichment media in combination with assay performance for detecting *Campylobacter* in food matrices. Pre- and post-harvest poultry research on *Campylobacter* and *Salmonella* have primarily relied on culture-based methods such as ISO 10272 [15,26,27,28]. This study aimed to develop and optimize a more rapid quantification method for *Campylobacter* (CampyQuant™) in post-chill poultry WBCR using a shortened enrichment-based quantification. The authors adapted a commercialized assay, the BAX^®^ System Real-Time PCR *Campylobacter* assay, to be utilized as an enrichment-based quantification method. 

### 4.1. Objective 1: Determining Optimal Enrichment Media and Enumeration Range of BAX^®^ System Real-Time PCR Assay

In the present study, two current industry standard media, 2× BFBB and BPW, were compared to determine the detection parameters of the BAX^®^ System Real-Time PCR assay, as well as determine the best-fit media for enrichment-based PCR quantification. The results of objective 1 indicated that the BAX^®^ System could detect *Campylobacter* in both respective media, but the optimal media was 2× BFBB. These results are in congruence with the ISO 10272-1:2017 method for detecting *Campylobacter* spp. in food [26], which also recommends using 2× BFBB. It was determined in the current study that WBCR enriched in 2× BFBB had the lowest detection limit for all *Campylobacter* species in the BAX^®^ System Real-Time PCR assay (*C. jejuni*: 3.03; *C. coli*: 2.93; *C. lari*: 3.13 Log_10_ CFU/mL). Main effect differences between media 2× BFBB and BPW were observed for all quantification metric parameters analyzed. When the full model was examined, an interaction among *Campylobacter* species and the media was observed, with 2× BFBB resulting in greater accuracy than BPW in both *C. jejuni* and *C. coli*. Similar results were seen in a study by Solis-Soto et al. [29]. Solis-Soto et al. [29] used various enrichment broths, including Preston’s, Bolton’s broth, Blood-Free Enrichment Broth (BFEB), and Modified-BFEB (M-BFEB) to determine the recovery rate of *Campylobacter* spp. Solis-Soto et al. [29] indicated that *Campylobacter* could be recovered from all enrichment broths; however, Bolton’s broth resulted in the greatest detection sensitivity by consistently allowing detection as low as 10 *C. jejuni* cells. *Campylobacter* cell recovery was followed by BFEB, M-BFEB, and Preston, with the lowest sensitivity of 10^3^ cells [29]. 

As BPW is a non-selective media, it has sufficient nutrients to allow multiple organisms present to grow. However, it is possible that non-neutralized antimicrobials used throughout processing can appear in the rinsates and subsequently interfere with *Campylobacter* recovery [21]. Moreover, competition between competing organisms during enrichment will likely inhibit *Campylobacter* growth. Therefore, the current study suggests that 2× BFBB be used as the optimal medium for *Campylobacter* detection. Using 2× BFBB as the enrichment broth, the BAX^®^ System Real-Time PCR assay appeared to be highly specific for each primer set for the detection of *C. jejuni*, *C. coli*, and *C. lari*, with 100% specificity for each. Therefore, 2× BFBB was used for the enrichment time of detection and validation studies to remain consistent with industry standards [20].

### 4.2. Objective 2: Time of Detection of Campylobacter spp. in Enriched Poultry WBCR

Although interventions are in place to reduce *Campylobacter* and other foodborne pathogens, they are not entirely eliminated. Broiler chickens, a natural reservoir for *Campylobacter*, typically harbor 10^5^ to 10^8^ in their gastrointestinal tract when harvested for processing [7]. Currently, there are no *Campylobacter* detection limitations on poultry products in the United States. However, the European Union (EU) allows less than 3.00 Log_10_ CFU/g of *Campylobacter* on poultry carcasses [30]. For the development of quantitation parameters, 3.00 Log_10_ CFU/mL was considered the detection limit of *Campylobacter* in poultry rinsates in the current study. Therefore, a pre-enrichment step is needed to detect and quantify the pathogen. As a result, further research was performed to determine the appropriate time of enrichment for low-level *Campylobacter* to grow sufficiently to reach the level of detection in the BAX^®^ System Real-Time PCR. The enrichment times in the current study were based on the growth kinetics of *C. jejuni*, *C. coli*, and *C. lari* pure cultures enriched in 2× BFBB [24]. Therefore, the current research investigated the enrichment time needed during the exponential phase for all three species to exceed the limit of detection in the presence of WBCR indigenous microbiota.

In poultry WBCR inoculated with each *Campylobacter* spp., the detection limit was 3.90, 4.50, and 3.80 Log_10_ CFU/mL in the BAX^®^ System Real-Time PCR assay for *C. jejuni*, *C. coli*, and *C. lari*, respectively. The detection limit of the BAX^®^ System Real-Time PCR assay was less effective for poultry WBCR samples than for pure culture (*C. jejuni*: 3.03; *C. coli*: 2.93; *C. lari*: 3.13 Log_10_ CFU/mL). The higher detection limit of poultry WBCR is likely because foodborne pathogens such as *Campylobacter* are generally in lower abundance on processed poultry carcass microbiota and thus require propagation for detection. In addition, competitor organisms, loss of template during DNA extraction, and inhibitors present in the rinsate could reduce the detection efficiency [31]. Similar results were seen in a study by Zhang et al. [31]). Using their multiplex PCR for *Campylobacter* detection, they reported a limit of detection of 4.3 CFU/g in pure culture but 10^3^ CFU/g in cecal contents. Therefore, enrichment was necessary to enumerate low levels of *Campylobacter* species.

Additionally, the poultry rinsates consisted of a wide range of microorganisms, which could influence *Campylobacter* growth and biochemical interactions in media, potentially supporting the development of non-*Campylobacter* colonies. Ricke et al. [15] suggested that different poultry matrices could induce biochemical changes to *Campylobacter* and actively interfere with the sensitivity and specificity of certain isolation and detection methods. These results further support the difficulties associated with *Campylobacter* detection using either molecular-based or culture-based methods in complex matrices [15,31]. 

According to the USDA-FSIS [20] method of isolation, *Campylobacter* can require up to 48 h of enrichment before it is detectable. In this study, the BAX^®^ System Real-Time PCR assay for *Campylobacter* yielded 100% detection in all samples after 20 h incubation in poultry rinsates for concentrations of 2.00 Log_10_ CFU/mL (Table 2). Also, the performance characteristics of the assay improved as time progressed to 20 h, proving 20 h to be the optimal enrichment period to be utilized for the final validation of the BAX^®^ System Real-Time PCR *Campylobacter* assay.

### 4.3. Objective 3: Validation and Standard Curve Development of Quantification of BAX^®^ System Real-Time PCR Assay 

To validate the use of CampyQuant™ in poultry WBCR, the detected *Campylobacter* concentrations from the poultry WBCR samples and CT values from the BAX^®^ System Real-Time PCR assay were used to develop standard curves with an enumerable range of 1.00 to 4.00 Log_10_ CFU/mL. In poultry WBCR, the BAX^®^ System Real-Time PCR assay was 100% specific for the detection of *Campylobacter* levels of 1.00 to 4.00 Log_10_ CFU/mL after 20 h enrichment. The Log_10_ RMSE evaluates the standard deviation of the data and illustrates the utility of the CT values for estimating the respective *Campylobacter* concentrations. From these results, using CampyQuant™ for *C. jejuni*, *C. coli*, and *C. lari* quantification, there was a variation of approximately 0.64, 1.09, and 0.81 Log_10_ CFU/mL in rinsate samples quantified between 1.00 and 4.00 Log_10_ CFU/mL after 20 h enrichment, respectively. While the variability of each standard curve fluctuated, it is likely the variability of the spread plating, which is still considered an acceptable standard, as being potentially subject to phenotypic-based assumptions [32]. However, when evaluated, CampyQuant™ estimates with 95% confidence intervals reached the targeted inoculated levels of each *Campylobacter* species. As observed, the paired evaluation of the CampyQuant™ and the Campy Cefex plating method on inoculated samples produced comparable estimates, with confidence intervals bracketing the targeted inoculated levels of *Campylobacter*. 

Quantification parameters such as sensitivity, accuracy, prevalence, NLR, and NPV were evaluated between the quantification method (CampyQuant™ vs. Campy Cefex) and the *Campylobacter* species for main effects and interactions. The results from the study demonstrate that the BAX^®^ System Real-Time PCR assay for *Campylobacter* detection appears to be precise and accurate compared to the culture enumeration method, Campy Cefex (Table 3 and Table 4). Additionally, CampyQuant™ does lend some advantages for *Campylobacter* detection and quantification. Some physiological and metabolic biological characteristics of *Campylobacter* can be altered due to environmental stress, which can cause *Campylobacter* cells to be viable but nonculturable [33]. In the present study, it is possible that cells could be viable but nonculturable on Campy Cefex plates. This could lead to underestimations of *Campylobacter* colonies on Campy Cefex plates. However, these VBNC populations can be accounted for using the CampyQuant™ assay. 

In addition, the conventional culture method in this study is that selective media is not able to specify differences between different *Campylobacter* species just by visually examining the colony morphology. The specificity of the assay by the species-specific amplification of DNA was 100%, as all *Campylobacter* samples were detected as such in the present study. Moreover, the cells on the Campy Cefex plates may not be as specific as the RT-PCR. In a study by Kim et al. [34], they compared sequences from poultry carcass rinsates from Campy Cefex plates. The plate colonies recovered revealed a significant range of non-*Campylobacter* bacteria, such as *Oscillospira*, *Acinetobacter*, *Enterococcus*, and *Bacillus* [34]. The recovery of non-*Campylobacter* bacteria shows the potential for false positives to occur with some culture methods. 

Nevertheless, molecular methods for *Campylobacter* detection and quantification do have some concerns. Traditional DNA-based PCR is able to detect the genomic DNA of dead or nonviable *Campylobacter* spp. However, the detection of these organisms, which would be expected not to be capable of causing disease, should be considered false-positive samples [15]. However, the growth of viable cells will dominate the nonviable cells during enrichment. In addition, the free genomic DNA from the dead cells is denatured from the DNase enzymatic activity from the enriched *Campylobacter* cells [35,36]. This process allows for potential dead cells to be excluded from the detection signal of the PCR.

Slopes of species-specific CampyQuant™ curves were explored for significant differences. The ANCOVA test revealed a significant difference among the species of *Campylobacter* (F-Value of 0.023). Differences were expected because each *Campylobacter* species possesses unique metabolic requirements [37]. These unique metabolic characteristics of each *Campylobacter* species would be anticipated to have specific interactions with the poultry WBCR, which can alter the detection in the RT-PCR. For example, Wagley et al. [38] studied the different carbon substrate utilization patterns of *Campylobacter* species. It was revealed that *C. coli* and *C. jejuni* differed in their ability to utilize propionic acid as a carbon source in a culture medium.

Additionally, these differences in metabolism can also result in growth rate differences. In a study by Axelsson-Olsson et al. [39], they compared the growth rates of different *Campylobacter* species after 48 h. The study revealed that *C. jejuni*, *C. coli*, and *C. lari* exhibited significantly different exponential phases from each other [39]. The metabolic requirement and growth kinetic differences between species can cause the differences observed in the standard curve slopes. Therefore, each *Campylobacter* species should be quantified with its respective specific CampyQuant™ standard curve.

## 5. Conclusions

In summary, the BAX^®^ System Real-Time PCR assay detected *Campylobacter* in all post-chill rinsate samples through an enumerable range of 1.00 to 4.00 Log_10_ CFU/mL. Based on the results of the current study, 20 h 2× BFBB enriched WBCR samples can be quantified using the CampyQuant™ linear equation for *C. jejuni*, *C. coli*, and *C. lari*. All subsequent linear equations generated in the current study corresponded to the data, with an R^2^ of 0.934, 0.758, and 0.943, respectively. While *C. jejuni* and *C. lari* exhibited a strong association, *C. coli* was lower, which could lead to more errors in quantitative estimates for this species. However, the speed of the assay represents a sufficient enough advantage to still use this method in practical settings. The Log_10_ RMSE was also within 1 Log10 CFU/mL at 0.64, 1.09, and 0.81 Log_10_ RMSE. The linear equations generated to be used in the quantification of *C. jejuni* Equation (1), *C. coli* Equation (2), and *C. lari* Equation (3) are

$$C.\;jejuni\;\left({Log}_{10}\;CFU/mL\right)=\frac{CT-41.64}{-2.281}$$

$$C.\;coli\;\left({Log}_{10}\;CFU/mL\right)=\frac{CT-39.98}{-1.896}$$

$$C.\;lari\;\left({Log}_{10}\;CFU/mL\right)=\frac{CT-40.75}{-3.03}$$

Further validations of this method on enumerating *Campylobacter* spp. in commercial processing plants among post-chill WBCR containing a complex microbial background are a logical next step. The current study utilized bulk homogenate post-chill WBCR, which may represent potential limitations with different rinsate microbiota compositional profiles that may influence the quantitation parameters. Testing across different processing samples and conditions is likely needed to determine the ultimate utility of the method proposed in the current study. However, the development of linear equations generated in the current study is necessary for the further validation of the BAX^®^ System Real-Time PCR *Campylobacter* assay. Also, the linear equations generated from this work can only be utilized in WBCR as they were the only matrices validated in the current research. Due to the effect that individual matrices have on the specific growth rate of pathogens, whether this be indigenous microbiota or matrix composition, individual matrices would need to be validated separately to generate unique linear curves to be utilized in this enrichment-based quantification [40].

Ultimately, the linear equations generated from 20 h enrichment of WBCR in 2× BFBB will provide the industry with the necessary tools to easily and more rapidly quantify *C. jejuni*, *C. coli*, *and C. lari* without deviating from current industry or USDA-FSIS testing standards. Additionally, with the ability to differentiate and quantify individual *Campylobacter* species, further risk assessment studies can be conducted. This could also prove to be particularly useful for further examination of *Campylobacter* outbreaks occurring in other countries outside the United States [41,42,43]. This method is also aligned with current United States industry standards and can potentially provide the poultry industry with a more rapid and accurate quantitative method for *Campylobacter* enumeration to ensure that process controls are working adequately to provide safe products to consumers.

## Figures and Tables

**Table 1:** Main effect and interaction of the enrichment media and *Campylobacter* species between the sensitivity, accuracy, prevalence, negative likelihood ratio (NLR), negative predictive value (NPV), and specificity of CampyQuant™ BAX^®^ System Real-Time PCR assay ^1,2^.

	*C. jejuni*		*C. coli*		*C. lari*		Effects ^3^		
Media	2× BFBB	BPW	2× BFBB	BPW	2× BFBB	BPW	Interaction ^4^	Media	Species
Sensitivity (%)	93.3	76.0	96.3	64.0	88.0	80.0	0.102	<0.0001	0.277
Accuracy (%)	93.8 ^a^	81.9 ^bc^	96.3 ^a^	79.0 ^c^	89.8 ^ab^	84.0 ^bc^	0.024	<0.0001	0.855
Prevalence (%)	90.0	73.1	87.2	61.5	84.6	74.9	0.273	0.0022	0.366
NLR (%)	6.7	24.0	4.0	36.0	12.0	20.0	0.063	0.003	0.57
NPV (%)	38.9	14.5	55.6	11.2	27.9	16.7	0.312	0.009	0.578

^1^ Specificity, PLR, and PPV were 100% for all variables; therefore, statistical analyses were not performed. ^2^ Efficiency is excluded from analyses as it is a metric calculated over the duration of the experiment. ^3^ Significance was determined using the nonparametric Mann–Whitney U test (*p* ≤ 0.05). ^4^ Different letters denote significant pairwise differences (a–c) of means during the interaction of enrichment media and *Campylobacter* species using χ^2^ analysis (*p* ≤ 0.05).

**Table 2:** Main effect and interaction of the time and *Campylobacter* species on the sensitivity, accuracy, prevalence, negative likelihood ratio (NLR), and negative predictive value (NPV) of CampyQuant™ BAX^®^ System Real-Time PCR assay ^1,2^.

	*C. jejuni*			*C. coli*			*C. lari*			Effects		
Time	16 h	18 h	20 h	16 h	18 h	20 h	16 h	18 h	20 h	Interaction	Time	Species
Sensitivity	66.7 ^cd^	86.1 ^ab^	94.4 ^a^	14.6 ^e^	25.7 ^e^	63.9 ^cd^	13.9 ^e^	53.0 ^d^	72.2 ^bc^	0.0040	<0.0001	<0.0001
Accuracy	42.7 ^bc^	71.4 ^a^	78.1 ^a^	3.0 ^d^	8.6 ^d^	39.2 ^bc^	1.2 ^d^	27.3 ^c^	50.4 ^b^	0.0100	<0.0001	<0.0001
Prevalence	64.0	82.7	90.7	17.3	33.3	61.3	21.3	49.3	69.3	0.1610	<0.0001	<0.0001
NLR	33.3 ^bc^	13.9 ^de^	5.6 ^e^	85.1 ^a^	74.3 ^a^	36.1 ^bc^	86.1 ^a^	47.0 ^b^	27.7 ^cd^	0.0004	<0.0001	<0.0001
NPV	10.7 ^bc^	17.0 ^b^	44.4 ^a^	6.8 ^bc^	6.0 ^bc^	10.4 ^bc^	5.1 ^c^	6.8 ^bc^	12.6 ^bc^	<0.0001	<0.0001	<0.0001

^1^ Significance was determined using the nonparametric Mann–Whitney U test. ^2^ Different letters denote pairwise differences (a–e).

**Table 3:** Log_10_ CFU/mL estimates from Campy Cefex and CampyQuant™ for each species. Main effect and interactions of the quantification method (CampyQuant™ vs. Campy Cefex) and *Campylobacter* species on each specific Log_10_ CFU/mL estimates (1.00 to 4.00 CFU/mL). An ANCOVA test was used to investigate a significantly different linear slope between the species of *Campylobacter* (F-Value = 0.023).

	*C. jejuni*		*C. coli*		*C. lari*		Effects ^1^		
Log_10_ CFU/mL	CampyQuant™	Campy-Cefex	CampyQuant™	Campy-Cefex	CampyQuant™	Campy-Cefex	Interaction ^2^	Method	Species
1.00	1.18 ^b^ ± 0.16	1.64 ^a^ ± 0.16	1.22 ^ab^ ± 0.10	1.260 ^ab^ ± 0.12	1.18 ^b^ ± 0.16	0.70 ^c^ ± 0.10	0.002	0.913	0.001
2.00	1.89 ^bc^ ± 0.05	2.18 ^a^ ± 0.05	2.09 ^ab^ ± 0.06	1.74 ^c^ ± 0.05	1.88 ^bc^ ± 0.05	1.50 ^d^ ± 0.05	<0.001	<0.001	0.002
3.00	2.93 ^ab^ ± 0.04	3.05 ^ab^ ± 0.10	2.74 ^b^ ± 0.09	3.23 ^a^ ± 0.09	2.92 ^b^ ± 0.09	2.68 ^b^ ± 0.09	0.001	0.100	0.639
4.00	4.08 ^ab^ ± 0.08	4.03 ^ab^ ± 0.05	3.91 ^b^ ± 0.07	4.13 ^a^ ± 0.05	4.09 ^ab^ ± 0.07	3.55 ^c^ ± 0.05	<0.001	0.005	<0.001

^1^ Significance was determined using the nonparametric Mann–Whitney U test (*p* ≤ 0.05). ^2^ Means with different connecting letters (a–d) were significantly different in the interaction of method and *Campylobacter* species.

**Table 4:** Main effect and interaction of the quantification method (CampyQuant™ vs. Campy Cefex) and *Campylobacter* species on the sensitivity, accuracy, prevalence, negative likelihood ratio (NLR), and negative predictive value (NPV).

	*C. jejuni*		*C. coli*		*C. lari*		Effects ^1^		
Method	CampyQuant™	Campy-Cefex	CampyQuant™	Campy-Cefex	CampyQuant™	Campy-Cefex	Interaction	Method	Species
Sensitivity (%)	88.3	94.5	83.3	80.5	86.7	83.3	0.411	1.000	0.079
Accuracy (%)	74.7	82.5	66.5	60.0	71.7	64.5	0.415	0.7046	0.074
Prevalence (%)	84.1	87.2	79.4	74.3	82.5	76.9	0.433	0.405	0.083
NLR (%)	11.7	5.5	16.7	19.5	15.0	16.6	0.475	0.863	0.073
NPV (%)	34.4	30.5	20.6	15.9	26.1	27.8	0.733	0.527	0.020

^1^ Significance was determined using the nonparametric Mann–Whitney U test (*p* ≤ 0.05).

**Figure A1:**
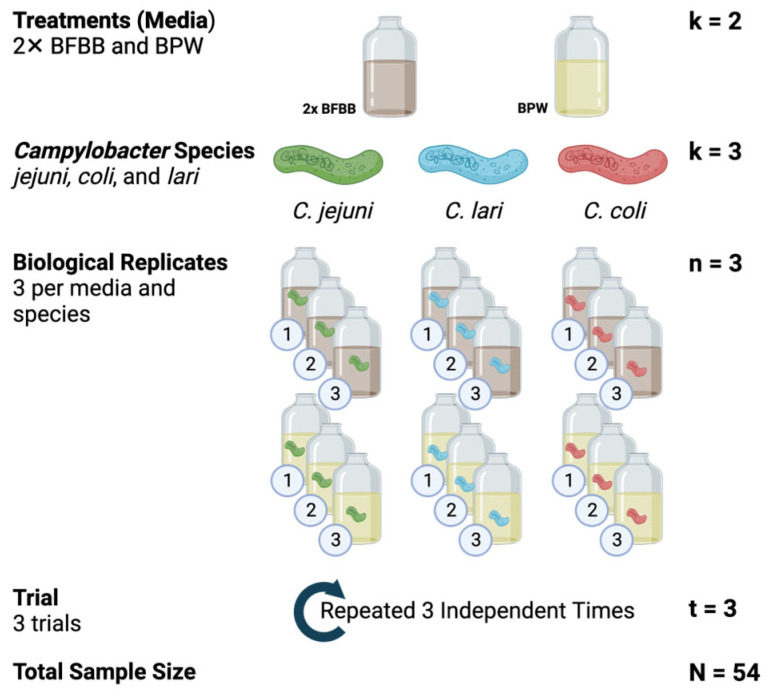
Representative schematic of Objective 1 experimental design and methodology. Created with Biorender.com.

**Figure A2:**
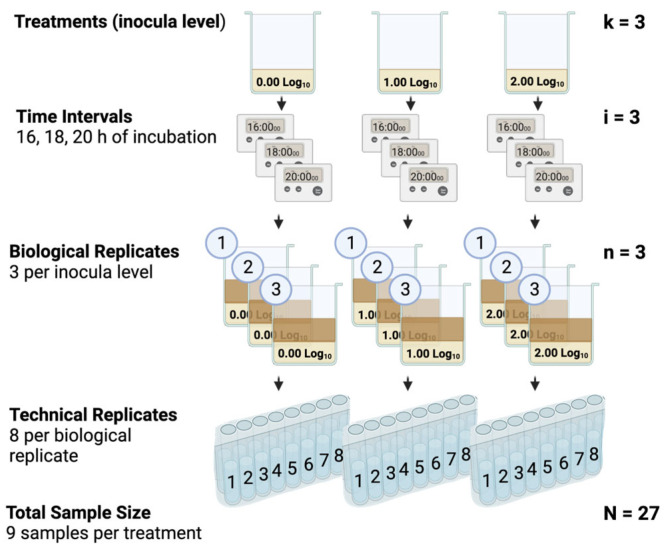
Representative schematic of Objective 2 experimental design and methodology. Created with Biorender.com.

## Data Availability

The data presented in this study are available on request from the corresponding author. The data are not publicly available due to the proprietary nature of the research.

## Supplementary Materials

The following supporting information can be downloaded at: https://www.mdpi.com/article/10.3390/foods13010056/s1, Table S1. The sensitivity, specificity, efficiency, positive likelihood ratio (PLR), negative likelihood ratio (NLR), prevalence, positive predictive value (PPV), negative predictive value (NPV), accuracy of the detection of *Campylobacter* species between media, blood-free Bolton broth (2× BFBB), and buffered peptone water (BPW) when using BAX^®^ System Real-Time PCR assay; Table S2. Statistical significance between the sensitivity, accuracy, prevalence, negative likelihood ratio (NLR), negative predictive value (NPV), and specificity between the species in either media, 2× blood-free Bolton broth (2× BFBB) or buffered peptone water (BPW); Table S3. Statistical significance between the sensitivity, accuracy, prevalence, negative likelihood ratio (NLR), negative predictive value (NPV), and specificity between the enrichment media, 2× blood-free Bolton broth (BB), or buffered peptone water (BPW) for *Campylobacter jejuni*, *coli*, and *lari*; Table S4. Plate counts of initial *Campylobacter* species concentration from growth and dilution in 2× Bolton broth and buffered peptone water on mCCDA; Table S5. Results of sensitivity, specificity positive likelihood ratio (PLR), negative likelihood ratio (NLR), prevalence, positive predictive value (PPV), negative predictive value (NPV), and accuracy of the BAX^®^ System Real-Time PCR assay for the detection of each *Campylobacter* species between media, enrichment times 16, 18, and 20 h in 2× blood-free Bolton broth (2× BFBB); Table S6. Statistical significance between the sensitivity, accuracy, prevalence, negative likelihood ratio (NLR), negative predictive value (NPV) between the time (16, 18, and 20 h) of *Campylobacter jejuni*, *coli*, and *lari*; Table S7. Statistical significance between the sensitivity, accuracy, prevalence, negative likelihood ratio (NLR), negative predictive value (NPV) between *Campylobacter* spp., *jejuni*, *coli*, and *lari*, at 16, 18, and 20 h of enrichment; Table S8. Statistical comparison of CampyQuant™ vs. Campy Cefex of each Log concentration (1.00 to 4.00 Log_10_ CFU/mL) within each species (*C*. *jejuni*, *coli*, and *lari*); Table S9. Results of quantification metric parameters: sensitivity, specificity, positive likelihood ratio (PLR), negative Likelihood ratio (NLR), prevalence, positive predictive value (PPV), negative predictive Value (NPV), and accuracy of the BAX^®^ System Real-Time PCR assay for the detection of each *Campylobacter* species; Table S10. Statistical comparison of performance criteria between the species of *Campylobacter* (*C. jejuni*, *coli*, and *lari*) when using CampyQuant™ or Campy Cefex; Table S11: Statistical comparison of performance criteria between the *Campylobacter* quantification methods utilized in the current study, CampyQuant™ vs. Campy Cefex, used to detect *C*. *jejuni*, *coli*, and *lari*.

## Appendix A

The optimal enrichment media and the enumeration range of BAX^®^ System Real-Time *Campylobacter* PCR assay were determined in the Objective 1 experiment (Figure A1). The experiment compared to industry standard media, 2× BFBB and BPW (media, k = 2), three *Campylobacter* species, *C. jejuni*, *C. coli*, and *C. lari*, (species, k = 3), three biological replicates (n = 3) with three independent trials (t = 3; N = 54). Created with Biorender.com.

Rinsate samples were prepared in Objective 2 to determine the time *Campylobacter* was detected (Figure A2). The experiment consisted of three time points, 16, 18, and 20 h (i = 3), three treatments (inoculation level, k = 3), three biological replicates, and eight technical replicates (n = 24) with 72 samples per timepoint (N = 216 total samples). The experimental conditions and sample numbers were consistent across *Campylobacter* species, *jejuni*, *coli*, and *lari*.
