# Supplementary material for: Adaptation of a Commercial Qualitative BAX® Real-Time PCR Assay to Quantify Campylobacter spp. in Whole Bird Carcass Rinses
Source: Foods. 2023 Dec 22;13(1):56. doi: 10.3390/foods13010056 (PMC10778266; doi:10.3390/foods13010056)
Supplement: Supplementary file 1 [file foods-13-00056-s001.zip › Table S1.pdf]

Table S1. The sensitivity, specificity, efficiency, Positive Likelihood Ratio (PLR), Negative Likelihood Ratio (NLR), Prevalence, Positive Predictive Value (PPV), Negative Predictive Value (NPV), Accuracy of the detection of *Campylobacter* species between media, Blood free Bolton broth (2× BFBB), and Buffered peptone water (BPW) when using BAX® System Real-Time PCR Assay[1,2].

Media		*C. jejuni*	*C. coli*	*C. lari*
2× BFBB	Sensitivity	93.0 % (88.0 to 99.0)	96.0 %(86.0 to 100.0)	88.0 % (71.0 to 100.0)
	Specificity	100.0 %	100.0 %	100.0 %
	Efficiency	121.0%	100.0 %	96.0%
	PLR	100.0 %	100.0 %	100.0 %
	NLR	6.7 %	4.0 %	12.0 %
	Prevalence	89.7 %	87.2 %	84.6 %
	PPV	100.0 %	100.0 %	100.0 %
	NPV	38.9 %	55.5 %	27.8 %
	Accuracy	94.0%	96.2%	89.4%
BPW	Sensitivity	76.0 % (66.0 to 86.0)	64.0 % (42.0 to 100.0)	80.0 % (80.0 to 100.0)
	Specificity	100.0 %	100.0 %	100.0 %
	Efficiency	107.0 %	371.0 %	93.0 %
	PLR	100.0 %	100.0 %	100.0 %
	NLR	24.0 %	36.0 %	20.0 %
	Prevalence	73.1 %	61.5 %	76.9 %
	PPV	100.0 %	100.0 %	100.0 %
	NPV	14.5 %	11.2 %	16.7 %
	Accuracy	81.8%	79.7%	84.0 %

[1]Significance for the main effect of species separated by media is presented in **Table S2**
[2]Significance for the main effect of media separated by *Campylobacter* species is presented in **Table S3**
