# Supplementary material for: Adaptation of a Commercial Qualitative BAX® Real-Time PCR Assay to Quantify Campylobacter spp. in Whole Bird Carcass Rinses
Source: Foods. 2023 Dec 22;13(1):56. doi: 10.3390/foods13010056 (PMC10778266; doi:10.3390/foods13010056)
Supplement: Supplementary file 1 [file foods-13-00056-s001.zip › Table S10.pdf]

Table S10. Statistical comparison of performance criteria between the species of *Campylobacter* (*C. jejuni*, *coli*, and *lari*) when using CampyQuant™ or Campy-Cefex.[1]

	CampyQuant™	CampyCefex
Sensitivity	P = 0.591	P = 0.089
Accuracy	P = 0.552	P = 0.090
Prevalence	P = 0.591	P = 0.089
NLR	P = 0.591	P = 0.079
NPV	P = 0.329	P = **0.049**

[1]Significance was determined using the nonparametric χ^2 analysis
