# Supplementary material for: Adaptation of a Commercial Qualitative BAX® Real-Time PCR Assay to Quantify Campylobacter spp. in Whole Bird Carcass Rinses
Source: Foods. 2023 Dec 22;13(1):56. doi: 10.3390/foods13010056 (PMC10778266; doi:10.3390/foods13010056)
Supplement: Supplementary file 1 [file foods-13-00056-s001.zip › Table S11.pdf]

Table S11. Statistical comparison of performance criteria between the *Campylobacter* quantification methods utilized in the current study, CampyQuant™ vs. Campy-Cefex, used to detect *C. jejuni*, *coli*, and *lari*.[1]

	C. jejuni	*C. coli*	*C. lari*
Sensitivity	P = 0.268	P = 0.376	P = 0.825
Accuracy	P = 0.825	P = 0.268	P = 0.825
Prevalence	P = 0.268	P = 0.268	P = 0.268
NLR	P = 0.289	P = 0.369	P = 0.825
NPV	P = 0.817	P = 0.268	P = 0.361

[1]Significance was determined using the nonparametric χ^2 analysis
