# Supplementary material for: Adaptation of a Commercial Qualitative BAX® Real-Time PCR Assay to Quantify Campylobacter spp. in Whole Bird Carcass Rinses
Source: Foods. 2023 Dec 22;13(1):56. doi: 10.3390/foods13010056 (PMC10778266; doi:10.3390/foods13010056)
Supplement: Supplementary file 1 [file foods-13-00056-s001.zip › Table S2.pdf]

Table S2. Statistical significance between the sensitivity, accuracy, prevalence, negative likelihood ratio (NLR), negative predictive value (NPV), and specificity between the species in either media, 2× blood-free Bolton broth (2× BFBB) or buffered peptone water (BPW).[1]

	2× BFBB	BPW
Sensitivity	P = 0.167	P = 0.074
Specificity	P = 1.000	P = 1.000
NLR	P = 0.167	P = 0.074
NPV	P = 0.459	P = 0.064
Prevalence	P = 0.549	P = 0.074
Accuracy	P = 0.167	P = 0.064

[1]Significance was determined using the nonparametric Mann-Whitney U test
