# Supplementary material for: Adaptation of a Commercial Qualitative BAX® Real-Time PCR Assay to Quantify Campylobacter spp. in Whole Bird Carcass Rinses
Source: Foods. 2023 Dec 22;13(1):56. doi: 10.3390/foods13010056 (PMC10778266; doi:10.3390/foods13010056)
Supplement: Supplementary file 1 [file foods-13-00056-s001.zip › Table S3.pdf]

Table S3. Statistical significance between the sensitivity, accuracy, prevalence, negative likelihood ratio (NLR), negative predictive value (NPV), and specificity between the enrichment media, 2× blood-free Bolton broth (BB), or buffered peptone water (BPW) for *Campylobacter jejuni*, *coli*, and *lari*.[1]

	C. jejuni	*C. coli*	*C. lari*
Sensitivity	P = **0.046**	P = **0.050**	P = 0.114
Accuracy	P = **0.046**	P = **0.050**	P = 0.114
Prevalence	P = **0.046**	P = 0.077	P = 0.114
NLR	P = **0.050**	P = 0.114	P = 0.114
NPV	P = 0.453	P = 0.101	P = 0.114
Specificity	P = 1.000	P = 1.000	P = 1.000

[1]Significance was determined using the nonparametric χ^2 analysis
