# Supplementary material for: Adaptation of a Commercial Qualitative BAX® Real-Time PCR Assay to Quantify Campylobacter spp. in Whole Bird Carcass Rinses
Source: Foods. 2023 Dec 22;13(1):56. doi: 10.3390/foods13010056 (PMC10778266; doi:10.3390/foods13010056)
Supplement: Supplementary file 1 [file foods-13-00056-s001.zip › Table S4.pdf]

Table S4. Plate counts of initial *Campylobacter* species concentration from growth and dilution in 2× Bolton broth and Buffered peptone water on mCCDA.[1]

	2× Bolton Broth	Buffered Peptone Water
C. jejuni	3.03 Log_{10} CFU/mL	5.60 Log_{10} CFU/mL
C. coli	2.93 Log_{10} CFU/mL	5.50 Log_{10} CFU/mL
C. lari	3.13 Log_{10} CFU/mL	4.50 Log_{10} CFU/mL
