# Supplementary material for: Adaptation of a Commercial Qualitative BAX® Real-Time PCR Assay to Quantify Campylobacter spp. in Whole Bird Carcass Rinses
Source: Foods. 2023 Dec 22;13(1):56. doi: 10.3390/foods13010056 (PMC10778266; doi:10.3390/foods13010056)
Supplement: Supplementary file 1 [file foods-13-00056-s001.zip › Table S5.pdf]

Table S5. Results of sensitivity, specificity Positive Likely Ratio (PLR), Negative Likely Ratio (NLR), Prevalence, Positive Predictive Value (PPV), Negative Predictive Value (NPV), and Accuracy of the BAX® System Real-Time PCR Assay for the detection of each *Campylobacter* species between media, enrichment times 16, 18, and 20 h in 2× Blood free Bolton broth (2× BFBB)[1,2].

Species	Inoculated Log_{10} CFU/mL	16 h	18 h	20 h
C. jejuni	0.58	18.0 %	73.0 %	93.0 %
	1.58	70.0 %	100.0 %	88.0 %
	2.58	100.0 %	100.0 %	100.0 %
	Sensitivity	66.7 %	86.1 %	94.4 %
	Specificity	100.0 %	100.0 %	100.0 %
	PLR	100.0 %	100.0 %	100.0 %
	NLR	33.3 %	13.9 %	5.60 %
	Prevalence	64.0 %	82.7 %	90.7 %
	PPV	100 %	100 %	100 %
	NPV	10.7 %	17.0 %	44.4 %
	Accuracy	42.7 %	71.2 %	85.6 %
C. coli	0.17	0.0 %	0.0 %	5.0 %
	1.17	0.0 %	5.0 %	92.0 %
	2.17	58.0 %	100.0 %	100.0 %
	Sensitivity	18.1 %	34.7 %	65.3 %
	Specificity	100.0 %	100.0 %	100.0 %
	PLR	100.0 %	100.0 %	100.0 %
	NLR	81.9 %	65.3 %	34.7 %
	Prevalence	17.3 %	33.3 %	61.3 %
	PPV	100.0 %	100.0 %	100.0 %
	NPV	6.81 %	6.00 %	10.4 %
	Accuracy	3.0%	8.6%	39.2 %
C. lari	0.03	0.0 %	0.0 %	21.0 %
	1.03	0.0 %	33.0 %	100 %
	2.03	42 %	63 %	100 %
	Sensitivity	13.9 %	31.9 %	72.2 %
	Specificity	100.0 %	100.0 %	100.0 %
	PLR	100.0 %	100.0 %	100.0 %
	NLR	86.1 %	68.1 %	27.8 %
	Prevalence	21.3 %	49.3 %	69.3 %
	PPV	100.0 %	100.0 %	100.0 %
	NPV	5.1 %	6.8 %	12.6 %
	Accuracy	1.2 %	27.3 %	50.4 %

[1]Significance for the main effect of time separated by *Campylobacter* species is presented in **Table S6**

[2]Significance for the main effect of *Campylobacter* species separated by time is presented in **Table S7**
