# Supplementary material for: Adaptation of a Commercial Qualitative BAX® Real-Time PCR Assay to Quantify Campylobacter spp. in Whole Bird Carcass Rinses
Source: Foods. 2023 Dec 22;13(1):56. doi: 10.3390/foods13010056 (PMC10778266; doi:10.3390/foods13010056)
Supplement: Supplementary file 1 [file foods-13-00056-s001.zip › Table S6.pdf]

Table S6. Statistical significance between the sensitivity, accuracy, prevalence, negative likelihood ratio (NLR) negative predictive value (NPV) between the time (16, 18, and 20 h) of *Campylobacter jejuni*, *coli*, and *lari*.[1]

	C. jejuni	*C. coli*	*C. lari*
Sensitivity	P = **0.045**	P = **0.034**	P = **0.027**
Accuracy	P = **0.027**	P = **0.034**	P = **0.027**
Prevalence	P = **0.027**	P = **0.034**	P = **0.038**
NLR	P = **0.027**	P = **0.034**	P = **0.027**
NPV	P = **0.033**	P = 0.102	P = 0.061

[1]Significance was determined using the nonparametric χ^2 analysis
