# Supplementary material for: Adaptation of a Commercial Qualitative BAX® Real-Time PCR Assay to Quantify Campylobacter spp. in Whole Bird Carcass Rinses
Source: Foods. 2023 Dec 22;13(1):56. doi: 10.3390/foods13010056 (PMC10778266; doi:10.3390/foods13010056)
Supplement: Supplementary file 1 [file foods-13-00056-s001.zip › Table S7.pdf]

Table S7. Statistical significance between the sensitivity, accuracy, prevalence, negative likelihood ratio (NLR) negative predictive value (NPV) between the *Campylobacter* spp., *jejuni*, *coli*, and *lari*, at 16, 18, and 20 h of enrichment.[1]

	16 h	18 h	20 h
Sensitivity	P < 0.054	P = 0.061	P = **0.030**
Accuracy	P < 0.059	P = **0.047**	P = **0.027**
Prevalence	P = 0.050	P = 0.061	P = **0.006**
NLR	P = 0.054	P = 0.062	P = **0.030**
NPV	P = 0.840	P = 0.060	P = **0.030**

[1]Significance was determined using the nonparametric χ^2 analysis
