# Supplementary material for: Adaptation of a Commercial Qualitative BAX® Real-Time PCR Assay to Quantify Campylobacter spp. in Whole Bird Carcass Rinses
Source: Foods. 2023 Dec 22;13(1):56. doi: 10.3390/foods13010056 (PMC10778266; doi:10.3390/foods13010056)
Supplement: Supplementary file 1 [file foods-13-00056-s001.zip › Table S8.pdf]

Table S8. Statistical comparison of CampyQuant™ vs. Campy-Cefex of each Log concentration (1.00 to 4.00 Log_{10} CFU/mL), within each species (*C. jejuni*, *coli*, and *lari*).[1]

Log_{10} CFU/mL	*C. jejuni*	*C. coli*	*C. lari*
1.00	**P = 0.001**	P = 0.469	**P < 0.001**
2.00	P = 0.173	P = 0.590	**P < 0.001**
3.00	**P < 0.001**	P = 0.061	P = 0.092
4.00	P = 0.739	P = 0.057	P = 0.230

[1]Significance was determined using the nonparametric χ^2 analysis
