# Supplementary material for: Adaptation of a Commercial Qualitative BAX® Real-Time PCR Assay to Quantify Campylobacter spp. in Whole Bird Carcass Rinses
Source: Foods. 2023 Dec 22;13(1):56. doi: 10.3390/foods13010056 (PMC10778266; doi:10.3390/foods13010056)
Supplement: Supplementary file 1 [file foods-13-00056-s001.zip › Table S9.pdf]

Table S9. Results of quantification metric parameters: sensitivity, specificity Positive Likely Ratio (PLR), Negative Likely Ratio (NLR), Prevalence, Positive Predictive Value (PPV), Negative Predictive Value (NPV), and Accuracy of the BAX® System Real-Time PCR Assay for the detection of each *Campylobacter* species.

Method	Performance Test	*C. jejuni*	*C. coli*	*C. lari*
CampyQuant™	Sensitivity	88.3 %	83.3 %	86.6 %
	Specificity	100.0 %	100.0 %	100.0 %
	Positive Likely Ratio	100.0 %	100.0 %	100.0 %
	Negative Likely Ratio	11.7 %	16.7 %	13.3 %
	Prevalence	84.1 %	79.4 %	82.5 %
	PPV	100.0 %	100.0 %	100.0 %
	NPV	34.4 %	20.6 %	24.4 %
	Accuracy	74.1 %	66.1%	71.5 %
Campy-Cefex	Sensitivity	94.4 %	80.6 %	83.3 %
	Specificity	100.0 %	100.0 %	100.0 %
	Positive Likely Ratio	100.0 %	100.0 %	100.0 %
	Negative Likely Ratio	5.6 %	19.4 %	16.7 %
	Prevalence	87.2 %	74.4 %	76.9 %
	PPV	100.0 %	100.0 %	100.0 %
	NPV	30.6 %	30.6 %	27.7 %
	Accuracy	82.5%	60.0%	64.5%

[1]Significance for the main effect of *Campylobacter* species separated by quantification method is presented in **Table S10**

[2]Significance for the main effect of the quantification method separated by *Campylobacter* species is presented in **Table S11**
